# Employee Growth Mindset and Innovative Behavior: The Roles of Employee Strengths Use and Strengths-Based Leadership

**DOI:** 10.3389/fpsyg.2022.814154

**Published:** 2022-06-20

**Authors:** Qiang Liu, Yuqiong Tong

**Affiliations:** School of Economics and Management, Liaoning University of Technology, Jinzhou, China

**Keywords:** growth mindset, innovative behavior, strengths use, strengths-based leadership, workplace

## Abstract

This study aimed to investigate the relationship of employee growth mindset with innovative behavior and the mediating role of use of strength as well as the moderating role of strengths-based leadership in this relationship. Data with a sample of 244 employees working in diverse Chinese organizations were collected at two points in time. Results of bootstrapping analyses demonstrated that growth mindset is positively related to innovative behavior, employee strengths use partially mediates the positive relationship of growth mindset with innovative behavior, and strengths-based leadership strengthens the direct relationship between employee growth mindset and innovative behavior and the indirect relationship of employee growth mindset with innovative behavior *via* strengths use. This study advances growth mindset and innovative behavior theories and research.

## Introduction

Mindset theory, which originates from educational psychology, has attracted considerable interest of the researchers due to its positive effect on students' motivation and achievement (Yeager and Dweck, 2020; Xu et al., 2021). Dweck (2006) suggested that individual mindsets can be divided into two categories, namely, growth mindset, and fixed mindset. Individuals with a growth mindset believe that their attributes such as intelligence are malleable, whereas individuals endorsing a fixed mindset believe that their attributes are stable (Yeager and Dweck, 2020). Many studies have indicated that people with a growth mindset are more likely to learn from their mistakes and reach higher levels of learning performance and achievement than people with a fixed mindset (e.g., Asbury et al., 2016; Bostwick and Becker-Blease, 2018; Yeager and Dweck, 2020). In addition, previous research has also found that employee growth mindset contributes to improved employee engagement (Keating and Heslin, 2015), task performance, job satisfaction, and organizational citizenship behavior (Han and Stieha, 2020).

Unfortunately, we have less knowledge of the relationship between employee growth mindset and innovative behavior. Innovative behavior has been defined as “an employee's intentional introduction or application of new ideas, products, processes, and procedures to his or her work role, work unit, or organization” (Yuan and Woodman, 2010, p. 324); it is a crucial influencing factor of organizational effectiveness and sustainable development (Scott and Bruce, 1994; Aryee et al., 2012).

To motivate employees to exhibit more innovative behaviors, researchers have identified many antecedents to innovative behavior from the perspective of employee characteristics such as creative self-efficacy (Newman et al., 2018), proactive personality (Li et al., 2017), conscientiousness, and openness to experience (George and Zhou, 2001). However, to the best of our knowledge, no prior research has been found to empirically investigate the relationship between employee growth mindset and innovative behavior. In essence, innovative behavior is characterized by risk and difficulty (Janssen et al., 2004; Menguc and Auh, 2010). As employees with a growth mindset do not worry about making mistakes and are good at addressing issues (Chao et al., 2017), it is possible to postulate that employee growth mindset is positively related to innovative behavior. Thus, the first aim of this study is to test this relationship.

In addition, although prior literature has investigated the effects of growth mindset from diverse perspectives (e.g., Corradi et al., 2019; Cutumisu, 2019; Yeager et al., 2019; Wang et al., 2021), we have less knowledge about why growth mindset can lead to various positive outcomes. A recent study has investigated the cognitive mechanism (i.e., reasoning ability) underlying the effect of growth mindset (Wang et al., 2020). However, it is worth noting that behavioral process is also an important perspective for explaining the effect of mindset (Meyers et al., 2020). Unfortunately, existing literature on growth mindset neglects this point. Strengths use, defined as the behaviors that individuals proactively leverage their own strengths in various contexts (Van Woerkom et al., 2016), might serve as a potential mediator between growth mindset and innovative behavior because employees who regard personal strengths as malleable (Jach et al., 2018) are more likely to play to their strengths at work so as to further develop their strengths, thereby leading to increased innovative behavior (Ding et al., 2021). As a result, the study's second aim is to investigate the behavioral process mechanism (i.e., employee strengths use) through which growth mindset is positively related to innovative behavior.

More importantly, the efficacy of individual characteristics is influenced by the contextual factors (Orvis and Leffler, 2011). For instance, Tierney et al. (1999) found that employees high in adaptive cognitive style can execute more invention disclosure forms when the relationship between employee and supervisor is supportive and of high quality. In a similar vein, when a supervisor executes more strengths-based leadership behaviors, his/her followers with growth mindset will be likely to capitalize on their strengths at work, thereby leading to increased innovative behavior. Strengths-based leadership refers to the extent to which leaders take various actions to promote their own and employees' strengths identification, deployment, and development (Burkus, 2011). Strengths-based leadership conveys an important cue to employees that leveraging strengths at work is appreciated and encouraged by the employer organizations or leaders (Ding and Yu, 2021). According to trait activation theory, when a situation relevant to a trait provides cues for the display of trait-related behaviors, individuals will exhibit more relevant behaviors (Tett et al., 2021). Based on this logic, we can postulate that when strengths-based leadership is high, employees who have higher levels of growth mindset may exhibit more strengths use behaviors and subsequently execute more innovative behaviors. Therefore, the third aim of this study is to test the positively moderating effect of strengths-based leadership on the relationships between growth mindset, strengths use, and innovative behavior.

In sum, this study aimed to develop and examine a moderated mediation model regarding growth mindset, strengths-based leadership, strengths use, and innovative behavior. This study adds to growth mindset and innovative behavior literature in three ways. First, this is the first study to empirically investigate the relationship between employee growth mindset and innovative behavior, which provides new insight into the antecedent to innovative behavior, and extends previous research on the effect of growth mindset. Second, by examining the mediating effect of employee strengths use, this study contributes to a deeper understanding of the probable behavioral mechanism through which growth mindset is positively associated with innovative behavior. Third, by investigating the moderating effect of strengths-based leadership, this study helps find a better way of maximizing the effects of growth mindset in terms of enhanced strengths use and innovative behavior.

This study is organized as follows. We reviewed relevant literature and develop the hypotheses of this study in the “Theory and hypothesis development.” The “Method” section presents participants, data collection procedures, and measures. In the “Results” section, we displayed the results of confirmatory factor analyses, descriptive statistics, and hypotheses testing. The “Discussion” section explains the theoretical and practical implications, potential limitations, and directions for future research. Finally, we summarized this study in the “Conclusion” section.

## THEORY AND HYPOTHESES

### Growth Mindset and Innovative Behavior

Over the past two decades, mindset research has gradually triggered researchers' interest (e.g., Caniëls et al., 2018) in that mindset dominates our ways of perceiving the world and then influences our attitudes, motivation, and behaviors (Cseh et al., 2013). Growth mentality and fixed mindset are the two types of mindset (Yeager and Dweck, 2020). Importantly, in recent years, researchers have paid more attention to growth mindset because growth mindset can bring out more benefits to individuals such as increased intrinsic motivation compared with fixed mindset (Zhao et al., 2018) and decreased perceived cognitive load (Xu et al., 2021). Dweck (2006) has demonstrated that individuals with a growth mindset consider their characteristics such as talents, intelligence, strengths, and abilities as malleable. In the face of difficulties and setbacks, individuals high in growth mindset are more optimistic and resilient (Blackwell et al., 2007). Moreover, there was evidence that growth mindset is related to adaptive health and psychosocial outcomes such as lower anxiety and postoperative pain (Kain et al., 2021). Although several studies have also shown that growth mindset is able to lead to various desired outcomes for employees such as increased work engagement (Keating and Heslin, 2015; Zeng et al., 2019) and decreased work stress (Zhao and Chen, 2021), less is known about the relationship between employee growth mindset and innovative behavior.

This study postulates that growth mindset employees will execute more innovative behaviors. Employees with a growth mindset are more likely to view challenges and difficulties as crucial opportunities to learn and progress, according to the research (Paunesku et al., 2015; Chao et al., 2017). As innovative behavior is challenging and risky (Yuan and Woodman, 2010; Hsu and Chen, 2017), employees with a growth mindset might execute more innovation at work so as to learn from the process of innovation. Furthermore, growth mindset employees always work hard (Bedford, 2017), proactively seek feedback and help from others (Cutumisu, 2019), and try novel strategies to attain their goals (Abernethy et al., 2021). These positive behaviors not only contribute to employee innovation but also are the manifestation of employee innovative behavior. More importantly, O'Keefe et al. (2018) have pointed out that growth mindset might have a positive relationship with innovation. As a result, we suggested the following hypothesis, based on the foregoing rationale and the argument by O'Keefe et al. (2018).

Hypothesis 1: Employee growth mindset is positively related to innovative behavior.

### The Mediating Role of Employee Strengths Use

Alongside the development of positive psychology, strengths-based approaches have garnered more and more attention from scholars and practitioners (e.g., Proctor et al., 2011; Ruch et al., 2020). Therein, a growing body of research has focused on employee strengths use due to its positive effect on employees' attitudes, motivation, emotions, behaviors, and performance (e.g., Bakker and Van Woerkom, 2018; Bakker and van Wingerden, 2021). Employees who capitalize on strengths at work, for example, are more engaged at work and experience higher levels of work meaningfulness and job satisfaction (Littman-Ovadia et al., 2017). In addition, strengths use has been found to be positively related to wellbeing, self-esteem, and self-efficacy (Proctor et al., 2011), and be negatively associated with feelings of depression and stress (Wood et al., 2011; Huber et al., 2017). More importantly, when employees utilize their strengths at work, they are more apt to exhibit more innovative behaviors because strengths use can foster employees' positive affect (Ding et al., 2021); such emotional resource subsequently stimulates employees to take risky behaviors (Isen and Patrick, 1983), thereby promoting employee innovative behavior. Recent empirical research has provided evidence for the positive relationship between employee strengths use and innovative behavior (Ding et al., 2021).

Given that strengths use can lead to various positive outcomes, several scholars have investigated the antecedents to employee strengths use. Extant research found that individuals' characteristics such as core self-evaluation (Ding and Lin, 2020), proactive personality (Yi-Feng Chen et al., 2021), and strengths endorsement (Tang et al., 2019) contribute to enhanced strengths use. Nevertheless, we have yet to know whether growth mindset as a crucial individual characteristic (Mesler et al., 2021) relates to strengths use. This study believes that growth mindset is positively related to strengths use because individuals with a growth mindset tend to consider their strengths as malleable and are more likely to deploy their strengths in various positive ways (Jach et al., 2018). More importantly, Zhao et al. (2021) suggested that growth mindset is able to lead to valuable outcomes through behavioral mechanisms. Based on this argument, we postulated that growth mindset can positively influence innovative behavior *via* strengths use. In sum, the following hypothesis was derived.

Hypothesis 2: Employee strengths use mediates the relationship between employee growth mindset and innovative behavior.

### The Moderating Effect of Strengths-Based Leadership

Although strengths-based leadership has been demonstrated to be quite effective in promoting employee strengths use (Ding and Yu, 2021), we have yet to know whether it can act as a moderator between employee growth mindset and strengths use. This study postulates that strengths-based leadership can also enhance the positive relationship between employee growth mindset and strengths use. As demonstrated earlier, according to trait activation theory, when a situation relevant to a trait provides cues for a display of trait-related behaviors, individuals will exhibit more such behaviors (Tett et al., 2021). A great deal of empirical research has supported this argument (e.g., Zagenczyk et al., 2017; Luria et al., 2019). For instance, Javed et al. (2020) found that openness to experience will have a stronger influence on innovative work behavior when ethical leadership is high rather than low. Growth mindset can be treated as a specific strength-related trait (Ryazanov and Christenfeld, 2018). If a contextual factor conveys a signal to employees high in growth mindset that leveraging strengths at work is appreciated and encouraged, employees will tend to make the most of their strengths at work. Because substantial literature has shown that leadership can serve as an activation factor of traits in employees (e.g., Colbert and Witt, 2009; Xu and Yu, 2019), it is possible to anticipate strengths-based leadership as a moderator in the relationship between growth mindset and strengths use.

Specifically, when strengths-based leadership is high, employees can receive an important cue from leaders that their leaders appreciate and encourage employee strengths use (Ding et al., 2020). Based on the logic of trait activation theory, employees who hold a growth mindset will take advantage of their own strengths at work if their leaders exhibit more strengths-based leadership behaviors. This is because the signal that strengths-based leaders convey to employees contributes to activating strengths-related traits of employees. Accordingly, we believe that strengths-based leadership might enhance the positive relationship between growth mindset and strengths use. Furthermore, as strengths use might mediate the growth mindset-innovative behavior linkage, it is reasonable to believe that strengths-based leadership might boost the mediational effect of strengths use on the relationship between employee growth mindset and innovative behavior. Based on the above discussion, we postulated the following hypotheses.

Hypothesis 3. Strengths-based leadership positively moderates the relationship between growth mindset and strengths use in such a way that the relationship of growth mindset with strengths use is stronger when strengths-based leadership is high rather than low.

Hypothesis 4. Strengths-based leadership positively moderates the mediational effect of strengths use on the relationship between growth mindset and innovative behavior in such a way that the mediational effect of strengths use is stronger when strengths-based leadership is high rather than low.

**Figure 1:**
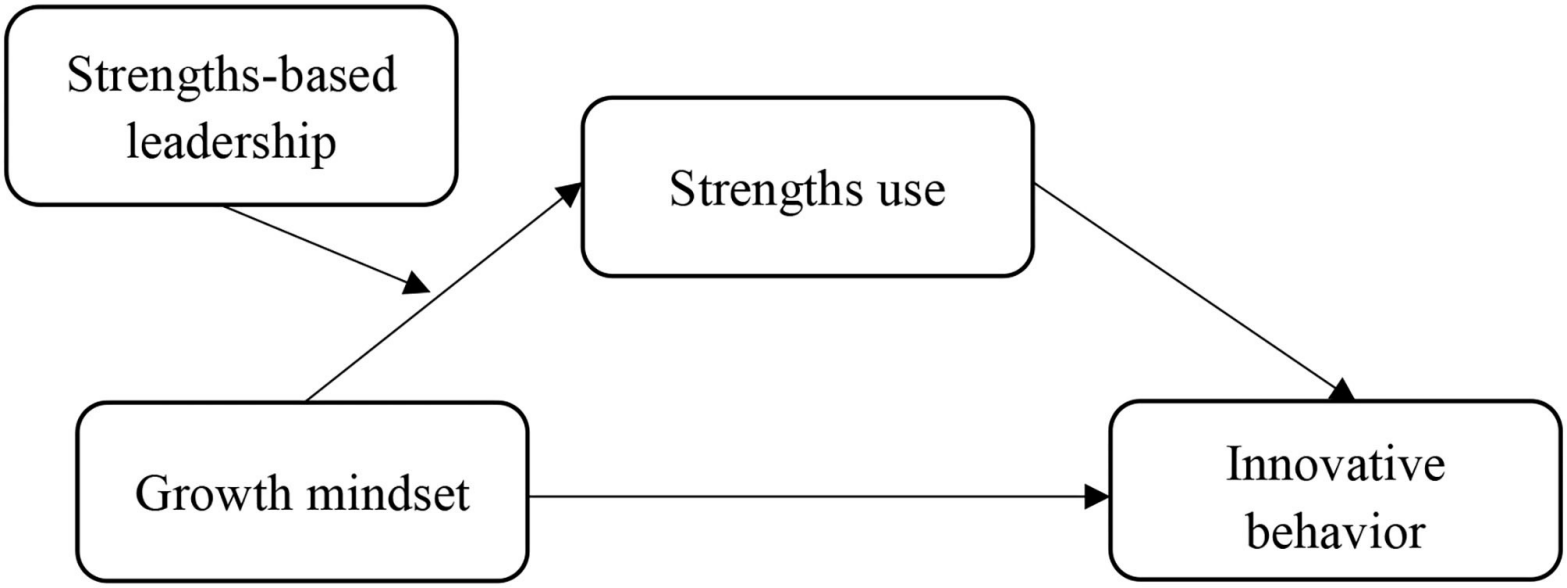
The research model.

The proposed research model is presented in Figure 1.

## Methods

### Research Approach

A questionnaire survey method was used for the current study. Due to its relatively low cost, the questionnaire survey method has been a popular method for data collection (Heeringa et al., 2017). A large number of extant studies have adopted this method to conduct empirical research (e.g., Rasool et al., 2022; Wang et al., 2022).

### Participants and Procedure

In this study, we adopted a convenience sampling method to recruit participants (Brewis, 2014). Self-administrated online questionnaires were applied to collect data. The first author invited 413 employees from various organizations (e.g., education industry and high-technology industry) in China through his social network to voluntarily participate in this study. We promised that the information about participants is only applied for academic research and kept confidential strictly. In addition, participants had the freedom to stop participating in this study at any time. To reduce common method variance (CMV), data were collected at two points in time, separated by a month interval. After receiving informed consent from the participants, we first distributed self-administrated online questionnaire comprising demographic variables, growth mindset scale, and strengths-based leadership scale to the participants. At Time 1, a total of 347 questionnaires were obtained (84.02% response rate). One month later, an online questionnaire regarding strengths use and innovative behavior scales was distributed to participants who responded at Time 1. We received 299 questionnaires (86.17% response rate) at Time 2.

After discarding ineffective data, which cannot be matched across two time points, we derived 244 valid matched data. Among them (see Table 1), 52.50% were women and 47.50% were men; 61.10% had achieved a bachelor's degree, 25.80% a master's degree, and 2.00% a doctor's degree. With respect to job level, 85.20% were general staff, 7.80% were front-line managers, 4.50% were middle-level managers, and 2.50% were the top-level managers. The average age of participants was 28.05 years (SD = 3.65), and the average tenure in the current organization was 3.10 years (SD = 3.03).

**Table 1:** Demographic characteristics.

**Characteristics**	**Category**	**Frequency**	**Percentage**
Gender	Male	116	47.54
	Female	128	52.46
Job level	Non-leader	208	85.25
	Front-line leader	19	7.79
	Middle leader	11	4.51
	Senior leader	6	2.46
Education	Specialist or under	27	11.07
	Bachelor	149	61.07
	Master	63	25.82
	Doctor	5	2.05

### Measures

#### Growth Mindset

We measured growth mindset with a four-item scale used by Kouzes and Posner (2019). Because the initial growth mindset scale was English-based edition, we obtained the Chinese edition of this scale following standard translation and back-translation procedures (Brislin, 1986). An example item was “Everyone, no matter who they are, can significantly change their basic characteristics.” Participants were required to rate these items on a seven-point Likert scale ranging from 1 (strongly disagree) to 7 (strongly agree). Furthermore, we also conducted exploratory factor analysis (EFA) to test scale's validity (Li, 2015). The result of EFA showed that the four-item scale explains 84.88% of the variance in growth mindset. The Cronbach's α of this scale was 0.94.

#### Strengths Use

Strengths use was evaluated with a Chinese five-item scale used by Ding et al. (2021). An example item was “In my job, I make the most of my strong points.” Participants were asked to rate these items on a five-point Likert scale ranging from 1 (strongly disagree) to 5 (strongly agree). The result of EFA showed that the five-item scale explains 81.85% of the variance in strengths use. The Cronbach's α of this scale was 0.94.

#### Strengths-Based Leadership

We evaluated strengths-based leadership with a Chinese eight-item scale developed by Ding et al. (2020). An example item was “My supervisor provides me with the opportunity to let me know what I am good at.” Participants were required to rate these items on a five-point Likert scale ranging from 1 (strongly disagree) to 5 (strongly agree). The result of EFA showed that the eight-item scale explains 70.46% of the variance in strengths-based leadership. The Cronbach's α of this scale was 0.94.

#### Innovative Behavior

Consistent with previous research (e.g., Nazir et al., 2019; Purc and Laguna, 2019), innovative behavior was measured with a six-item scale developed by Scott and Bruce (1994). As items of this scale were in English, we obtained the Chinese innovative behavior scale edition following standard translation and back-translation procedure (Brislin, 1986). An example item was “I generate creative ideas.” Participants were asked to rate these items on a seven-point Likert scale ranging from 1 (strongly disagree) to 7 (strongly agree). The result of EFA showed that the six-item scale explains 79.06% of the variance in innovative behavior. The Cronbach's α of this scale was 0.95.

## Results

### Confirmatory Factor Analysis

Confirmatory factor analysis was conducted to examine the discriminant validity of research variables. Analytical results are displayed in Table 2. The four-factor measurement model concerning growth mindset, strengths-based leadership, strengths use, and innovative behavior showed a better fit to the data than alternative measurement models, which indicated that these research variables have good discriminant validity.

**Table 2:** Confirmatory factor analyses of the research variables.

**Models**	**χ^2^**	**df**	**χ^2^/df**	**RMSEA**	**CFI**	**TLI**	**IFI**	**Δχ^2^(Δdf)**
Four-factor model (Baseline)	557.36	220	2.53	0.08	0.94	0.93	0.94	-
Three-factor model^a^	816.28	223	3.66	0.11	0.89	0.88	0.89	258.92^***^(3)
Two-factor model^b^	1,345.44	225	5.98	0.14	0.80	0.77	0.80	788.08^***^(5)
One factor model^c^	2,328.17	226	10.30	0.20	0.62	0.58	0.62	1,770.81^***^(6)

a *Growth mindset and strengths-based leadership combined.*

b *Growth mindset and strengths-based leadership combined, and strengths use and innovative behavior combined.*

c *All variables combined.*

*** *p < 0.001*.

Given that this study adopted a cross-sectional research design, we utilized the unmeasured common method factor method recommended by Podsakoff et al. (2003) to test the degree of CMV of research data. One common method factor was created and loaded on all items of growth mindset, strengths-based leadership, strengths use, and innovative behavior. Results of confirmatory factor analyses demonstrated that the five-factor measurement model comprising the common method factor and four research variables reports a better fit to the data (χ^2^ = 532.58, df = 219, χ^2^/df = 2.43, RMSEA = 0.08, CFI = 0.94, TLI = 0.94, IFI = 0.94) than the four-factor measurement model comprising four research variables, but the common factor merely elucidated 24.00% of variance, <25.00% (Williams et al., 1989). Hence, our study did not have severe CMV.

### Descriptive Statistics

Table 3 reports the means (M), standard deviations (SD), and correlational coefficients of research variables. Results of correlational analyses showed that growth mindset is positively related to strengths-based leadership (*r* = 0.43, *p* < 0.01), strengths use (*r* = 0.44, *p* < 0.01), and innovative behavior (*r* = 0.41, *p* < 0.01). In addition, strengths use was positively related to innovative behavior (*r* = 0.64, *p* < 0.01). These results provide initial evidence for our hypotheses.

**Table 3:** Means, standard deviations, and correlations.

**Variable**	**M**	**SD**	**1**	**2**	**3**
1. Growth mindset	4.99	1.21	-		
2. Strengths-based leadership	3.78	0.75	0.43^**^	-	
3. Strengths use	3.95	0.67	0.44^**^	0.31^**^	
4. Innovative behavior	5.07	1.21	0.41^**^	0.43^**^	0.64^**^

** *p < 0.01*.

### Hypothesis Testing

Multiple regression analysis with bootstrapping (5,000 re-sampling) was employed to test research hypotheses, and a 95% bias-corrected confidence interval was utilized to determine the significance of the regression coefficient. Hypothesis 1 postulated that growth mindset has a positive association with innovative behavior. As reported in Table 4 (Model 3), the coefficient of growth mindset was significant (β = 0.41, *p* < 0.001), supporting Hypothesis 1.

**Table 4:** Results for multiple regression analyses with bootstrapping.

**Variables**	**Strengths use**	**Innovative behavior**		
	**Model 1**	**Model 2**	**Model 3**	**Model 4**
Constant	2.73^***^	2.37^***^	3.04^***^	0.24
Growth mindset	0.24^***^	0.21^***^	0.41^***^	0.16^**^
Strengths use				1.02^***^
Strengths-based leadership		0.13		
Growth mindset × strengths-based leadership		0.08^*^		
*F*	57.88^***^	24.79^***^	47.83^***^	88.68^***^
*R* ^2^	0.19	0.24	0.17	0.42
Adjust-*R*^2^	0.19	0.23	0.16	0.42

*Unstandardized value was reported.*

*** *p < 0.001.*

** *p < 0.01.*

* *p < 0.05*.

Hypothesis 2 assumed that strengths use mediates the relationship between growth mindset and innovative behavior. As summarized in Table 4 (Model 4), the coefficient of strengths use was significant (β = 1.02, *p* < 0.001), and the coefficient of growth mindset was also significant (β = 0.16, *p* < 0.01). Thus, we could conclude that strengths use partially mediates the relationship between growth mindset and innovative behavior. In order to further test Hypothesis 2, Hayes's PROCESS (Model 4) with bootstrapping (5,000 re-sampling) was adopted. Results demonstrated that the indirect effect is significant [effect = 0.25, Boot SE = 0.05, 95% CI: (0.16, 0.36)]. Based on the above analyses, Hypothesis 2 received support from data.

**Figure 2:**
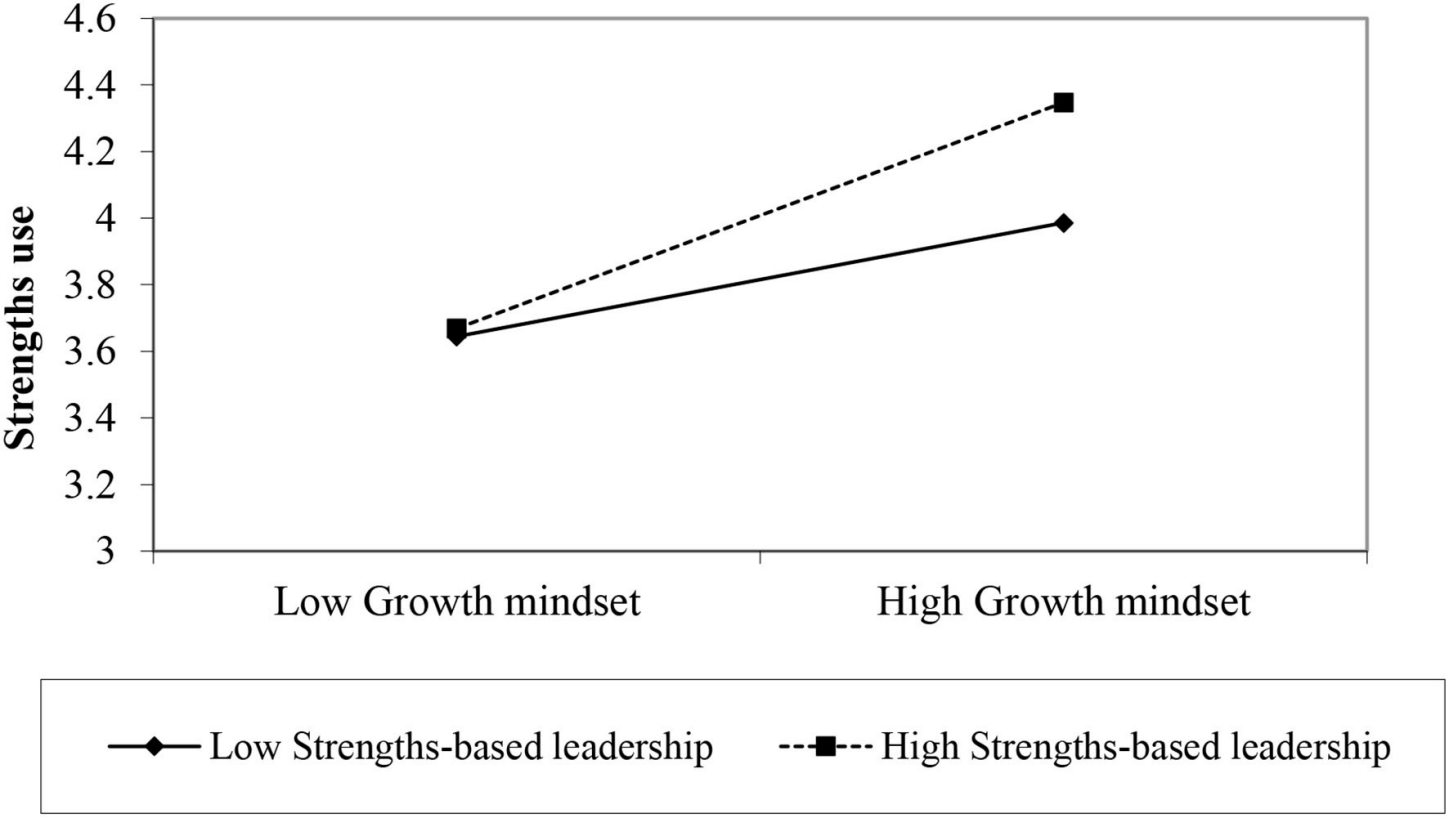
Plot of interactive effect.

Hypothesis 3 postulated that strengths-based leadership strengthens the relationship between growth mindset and strengths use. To examine this hypothesis, we first created the standardized values of growth mindset and strengths-based leadership, and then created the interaction term of growth mindset and strengths-based leadership. As displayed in Table 4 (Model 2), the interaction term was significant (β = 0.08, *p* < 0.05). To further test Hypothesis 3, Hayes's PROCESS (Model 1) with bootstrapping (5,000 re-sampling) was adopted. Analytical result showed that the interaction term is also significant [coefficient = 0.08, SE = 0.03, *t* = 2.85, *p* < 0.001, 95% CI: (0.03, 014)]. Slope analysis is depicted in Figure 2. Specifically, the conditional effect of growth mindset on innovative behavior is stronger when strengths-based leadership is high [M + 1 SD, effect = 0.34, SE = 0.05, *t* = 6.55, *p* < 0.001, 95% CI: (0.24, 0.44)] than low [M - 1 SD, effect = 0.17, SE = 0.05, *t* = 3.38, *p* < 0.001, 95% CI: (0.07, 0.27)]. Therefore, Hypothesis 3 received support.

Hypothesis 4 postulated that strengths-based leadership strengthens the indirect relationship of growth mindset with innovative behavior *via* strengths use. Hayes's PROCESS (Model 7) with bootstrapping (5,000 re-sampling) was adopted to inspect this claim. Result indicated that the index of moderated mediation is significant [index = 0.10, SE = 0.05, 95% CI: (0.01, 0.21)], and the mediational effect of strengths use is stronger when strengths-based leadership is high [M + 1 SD, effect = 0.29, SE = 0.06, 95% CI: (0.18, 0.42)] than low [M - 1 SD, effect = 0.14, SE = 0.06, 95% CI: (0.02, 0.28)]. Hence, Hypothesis 4 received support.

Finally, we conducted a *post-hoc* power analysis in G^*^Power with a sample size of 244 and three predictor variables as a baseline to inspect the appropriateness and representativeness of the research sample and findings. Consistent with Cohen's (1977) argument, we used three effect sizes (small, *f*_2_ = 0.02, medium, *f*_2_ = 0.15, large *f*_2_ = 0.35) for this evaluation. *Post-hoc* power analysis demonstrated that the power to detect the derived effect was 0.99 for the entire regression in prediction of employee innovative behavior at the 0.05 level beyond the value of 0.80 recommended by Cohen (1977). Accordingly, we believe that the power to detect small effects is enough with a sample of 244 and that the findings of this study are appropriate and representative.

## Discussion

This study of 244 employees working in various organizations in China investigated the relationship between growth mindset and innovative behavior and the mediational effect of strengths use as well as the moderating effect of strengths-based leadership in the relationship. All research hypotheses received support from research data. First, growth mindset is positively related to innovative behavior. This conclusion is in line with the argument by O'Keefe et al. (2018) that growth mindset contributes to individual innovation. Although several studies have explored the effects of growth mindset in working settings, to the best of our knowledge, this study is the first to empirically investigate the relationship of growth mindset with innovative behavior. The positive linkage between growth mindset and innovative behavior can be explained by the fact that employees high in growth mindset are more apt to try new strategies to attain goals (Abernethy et al., 2021) and to learn from mistakes and others' strengths (Dweck, 2014). In a word, this study enriches research on growth mindset in the workplace and provides new insight into the antecedent to innovative behavior.

Second, our study found that strengths use plays a vital mediating role in the growth mindset-innovative behavior linkage. This result is in line with the argument that growth mindset can result in various valuable outcomes through behavioral mechanisms (Zhao et al., 2021). Specifically, employees with a growth mindset are more inclined to treat strengths as changeable and then make the most of their strengths at work (Jach et al., 2018), thereby leading to increased innovative behavior. Although several scholars have proposed the behavioral mechanism through which growth mindset leads to desirable outcomes, this study is the first to provide empirical evidence for this proposition. Thus, the present study contributes to a better understanding of why growth mindset has a positive relationship with innovative behavior by revealing the mediating role of strengths use in the relationship.

Third, this study indicated that strengths-based leadership boosts the direct positive relationship between growth mindset and innovative behavior and the indirect relationship between growth mindset and innovative behavior *via* strengths use. This finding can be illuminated by trait activation theory suggesting that if a trait-related situation provides cues for display of trait-related behaviors, individuals are more likely to exhibit more relevant behaviors (Tett et al., 2021). Strengths-based leadership, in particular, offers a key signal to employees that their leaders encourage them to use their strengths at work. Such cue will motivate growth mindset employees to execute more strengths use behaviors that in turn executes more innovative behaviors. To the best of our knowledge, less research has explored the boundary condition of the effect of growth mindset in work settings. As such, this study addresses this gap and provides a vital way of optimizing the effect of growth mindset in terms of improved strengths use and innovative behavior.

### Practical Implications

The practical implications of this study are threefold. First, the positive relationship between growth mindset and innovative behavior implies that the employers can promote employees to carry out more innovative behaviors by cultivating employees' growth mindsets. Several methods are beneficial to fostering growth mindset of employees, such as implementing growth mindset training (Seaton, 2018) or a brief mindset intervention (Miller, 2019). Second, the mediational effect of strengths use on the relationship between growth mindset and innovative behavior denotes that the employers can also enhance employee innovative behavior by stimulating employees to use their strengths at work. For instance, promoting employee strengths development has been demonstrated to be positively correlated with employee strengths use (Biswas-Diener et al., 2011; Duan et al., 2019) have pointed out that helping individuals recognize their strengths is also conductive to boosting individual strengths use. Third, the positively moderating effect of strengths-based leadership on the relationships between growth mindset, strengths use, and innovative behavior indicates that the employers should shape strengths-based leadership to maximize the role of growth mindset in improving employee strengths use and innovative behavior. For example, training intervention has been confirmed to be an effective approach to shaping strengths-based leadership (Rath and Conchie, 2008; MacKie, 2014 have suggested that building a strengths-based culture is also able to cultivate strengths-based leadership.

### Limitations and Directions for Future Research

This study has four aspects of limitations. First, given the essence of the cross-sectional research design in this study, we adopted an appropriate method to exclude the serious threat of CMV to our results. Nevertheless, future research should also adopt longitudinal or experimental research to replicate our findings. Second, this study only investigated the behavioral mechanism underlying the effect of growth mindset. Parada and Verlhiac (2021) pointed out that growth mindset might lead to various valuable outcomes through the affective mechanism (e.g., positive affect). Future research should attempt to consider the affective mechanism through which growth mindset positively relates to innovative behavior. Third, according to the extant literature on strength-based approaches, strengths-based psychological climate and organizational support for strengths use similar to strengths-based leadership can also send an important signal to employees that employee strengths use is appreciated and encouraged by the organization (Van Woerkom et al., 2020; Moore et al., 2021). Future research should investigate whether other types of strengths-based approaches can also strengthen these relationships between growth mindset, strengths use, and innovative behavior. Fourth, concerning innovative behavior measure, a large number of scholars believe that it is better to evaluate employee innovative behavior by his/her supervisors or colleagues (e.g., Scott and Bruce, 1994; Newman et al., 2018). However, this study mainly investigated the relationship between growth mindset and self-perceived innovative behavior. Thus, future research should test employee growth mindset's relationship with other-rated innovative behavior.

## Conclusion

Although growth mindset has received considerable attention from researchers, less is known about whether employee growth mindset is related to innovative behavior. This study empirically investigated the relationship between employee growth mindset and innovative behavior. In addition, we also considered the mediating role of employee strengths use and the moderating role of strengths-based leadership in the relationship between employee growth mindset and innovative behavior. Research results indicated that employee growth mindset is positively related to employee innovative behavior, employee strengths use mediates the relationship between growth mindset and innovative behavior, and strengths-based leadership not only enhances the direct relationship between employee growth mindset and strengths use, but also strengthens the indirect relationship of growth mindset with innovative behavior *via* strengths use. This study advances employee growth mindset and innovative behavior theories and research.

## Data Availability Statement

The raw data supporting the conclusions of this article will be made available by the authors, without undue reservation.

## Ethics Statement

The studies involving human participants were reviewed and approved by Liaoning University of Technology. The patients/participants provided their written informed consent to participate in this study.

## Author Contributions

QL proposed the conceptual model, designed the research, and collected the research data. YT analyzed the data and wrote the manuscript. All authors contributed to the article and approved the submitted version.

## Funding

This research was funded by Research Base of Science and Technology Innovation Think Tank of Liaoning Province (Research Base of High Quality Development of Equipment Manufacturing Industry, No. 09) and 2021 Scientific Research Project of Department of Education of Liaoning Province (LJKR0225 and LJKR0224).

## Conflict of Interest

The authors declare that the research was conducted in the absence of any commercial or financial relationships that could be construed as a potential conflict of interest.

## Publisher's Note

All claims expressed in this article are solely those of the authors and do not necessarily represent those of their affiliated organizations, or those of the publisher, the editors and the reviewers. Any product that may be evaluated in this article, or claim that may be made by its manufacturer, is not guaranteed or endorsed by the publisher.

## APPENDIX

Growth mindset (Kouzes and Posner, 2019).

1. Everyone, no matter who they are, can significantly change their basic characteristics;
2. People can always substantially change the kind of person they are;
3. No matter what kind of person someone is, they can always change very much;
4. All people can change even their most basic qualities.

Strengths use (Ding et al., 2021).

1. In my job, I make the most of my strong points;
2. I organize my job to suit my strong points;
3. I capitalize on my strengths at work;
4. I seek opportunities to do my work in a manner that best suits my strong points;
5. In my job, I try to apply my talents as much as possible.

Strengths-based leadership (Ding et al., 2020).

1. My supervisor provides me with the opportunity to let me know what I am good at;
2. My supervisor encourages me to further develop my potential;
3. My supervisor is good at using my strengths;
4. My supervisor gives me more autonomy to use my strengths at work;
5. My supervisor discusses with me how I can improve my strengths;
6. My supervisor knows his or her talents;
7. My supervisor makes the most of his or her strong points at work;
8. My supervisor engages more his or her time and energy to develop his or her strengths.

Innovative behavior (Scott and Bruce, 1994).

1. I generate creative ideas;
2. I develop adequate plans and schedules for the implementation of new ideas;
3. I search out new technologies, processes, techniques, and/or product ideas;
4. I investigate and secure funds needed to implement new ideas;
5. I promote and champion ideas to others;
6. I am innovative.
